# Effect of ASF (a Compound of Traditional Chinese Medicine) on Behavioral Sensitization Induced by Ethanol and Conditioned Place Preference in Mice

**DOI:** 10.1155/2014/304718

**Published:** 2014-10-29

**Authors:** Da-chao Wen, Yi-bei Li, Xiao-Yu Hu, Wu Lin, Ling-yan Jia, Sen Zhong

**Affiliations:** ^1^Department of Clinical Medicine, Chengdu University of Traditional Chinese Medicine, Chengdu, Sichuan 610072, China; ^2^Department of Infectious Diseases, Affiliated Hospital of Chengdu University of Traditional Chinese Medicine, Chengdu, Sichuan 610072, China; ^3^Endocrinology Department, Affiliated Hospital of Chengdu University of Traditional Chinese Medicine, Chengdu, Sichuan 610072, China

## Abstract

ASF composed by semen and epimedium herbal is a traditional plant compound that is widely used in the treatment of insomnia. Studies have shown that saponins and flavonoids contained in semen can significantly decrease the content of excitatory neurotransmitter Glu in mice. And the total flavone of YinYangHuo can increase the release of GABA in the anterior periventricular system of rat and increase the affinity of GABA for the receptors GABAA. It can be inferred that their synergism may have effect on the neurotransmitter that causes behavioral sensitization and conditioned place preference in experimental animals and affects their drinking behaviors, which is the starting point of this research. The present study found that ASF can inhibit development and expression of behavioral sensitization induced by ethanol and the development of CPP in mice. We demonstrate the inhibition of ASF on behavioral sensitization partly due to its effect on the mesolimbic neurotransmitter system, including decreasing level of DA and Glu and increasing the content of GABA. It suggested that the ASF may have pharmacological effects in the treatment of alcohol addiction.

## 1. Introduction

Alcohol dependence is a kind of mental disorder characterized by compulsive drinking behavior, losing control over the intake of alcohol, and significant impairment of social and occupational function. According to WHO reported in 2004, the world had 2 billion drinkers, of which 140 million drinkers belong to the estimation of alcohol dependence. The annual number of deaths due to alcohol consumption leads to 1.8 million, accounting for 3.7% of the total number of deaths worldwide (including accidental injury deaths that accounted for one-third), total health costs $58.3 million, and accounting for 4.4% of the total burden of all diseases, including alcohol which leads to neuropsychiatric disorders that accounted for nearly 40% of disease burden. According to WHO reported in 2011, the number of harmful alcohol abuse deaths rose to 2.5 million a year, 6.2% of the world's male deaths related to alcohol; alcohol abuse is a risk, the third-largest global burden of disease. Currently, existing effective treatments for alcohol addiction is insufficient, although naltrexone for alcohol addiction can significantly reduce alcohol craving and alcohol consumption [1], digestion disorders, sleep disorders, liver damage, and other adverse reactions stay [2, 3]. When Fluoxetine is used for alcohol addiction, the compliance is poor and curative effect is not obvious [4]. Disulfiram and calcium cyanamide can cause a series of symptoms such as shortness of breath, facial flushing, headache, nausea, vomiting, palpitations, and even death [5–8] and poor compliance of patients. So it is necessary to look for a new temperance medicine.

Chinese herbal medicine has a potential therapeutic effect for alcohol addiction. According to Keung and other researchers [9–11], the extraction of isoflavones of daidzin and daidzein from Gegen (a Chinese herb) can effectively inhibit drinking behavior of hamsters. Guoyuan and other researchers [12] found that the Chinese herbal medicine decoction, JieJiuJieDuTang, could obviously delay the relapse time of drinking of alcohol dependent patients. Hong and other researchers [13] also found out that the effect of JieCheng oral liquid is good with nontoxic side effects. In China, SuanZaoRen and YinYangHuo are widely used as medicinal herbs that regulate mood and sleep. Studies have shown that saponins and flavonoids contained in SuanZaoRen can significantly decrease the content of excitatory neurotransmitter Glu in mice [14, 15]. And the total flavone of YinYangHuo can increase the release of GABA in the anterior periventricular system of rat and increase the affinity of GABA for the receptors GABAA [16]. It can be inferred that their synergism may have effect on the neurotransmitters that cause behavioral sensitization and conditioned place preference in experimental animals and affect their drinking behaviors, which is the starting point of this research.

Research shows that alcohol can induce behavioral sensitization [17], which can last a long time [18], and it has nothing to do with the sedative effects of alcohol [19]. Alcoholics and alcoholic's offspring have shown alcohol behavioral sensitization [20]. The above evidences indicated that behavioral sensitization plays an important role in the alcohol addiction. CPP is the classic experiment to determine material reward and spiritual dependence, as shown in the side change of natural preference after drug training. Morphine or amphetamine can induce CPP in rat. In this study, we use behavioral sensitization induced by ethanol and conditioned place preference in animal models to observe the influences of ASF on ethanol-induced behavioral sensitization and conditioned place preference, to evaluate the effects of ASF on the prevention and treatment of alcohol addiction, and to provide a theoretical basis of ASF in treating alcohol addiction.

## 2. Materials and Methods

### 2.1. Animals

Male Kunming mice of SPF level, 3 months old, weighing 22 ± 3 g, animal certificate number: SCXK (11) 2013-24, are provided by Chengdu Dashuo Animal Experimental Company. Laboratory illumination time is 7:00–19:00, the room temperature is 22–26°C, and humidity is 40–70%. Free drinking and eating are provided to mice. We try to reduce the number of mice used in experiments and to minimize the damage and pain of mice.

### 2.2. Drugs

ASF (YinYangHuo : SuanZaoRen = 4 : 5) specific composition is shown in Table 1. SuanZaoRen (batch no. 2013010506) and YinYangHuo (batch no. 2013062612) were purchased from Chengdu University of Traditional Chinese Medicine Affiliated Hospital Pharmacy. After the identification of professor Yan Zhuyun of Identification Department of the Chengdu University of TCM for genuine medicinal materials, the herbs were decocted, filtered, and concentrated to 1.5 g · mL^−1^. Saline, ethanol (concentration 96%), solution was prepared with saline (15%, v/v in 0.9% NaCl) and stored at 4°C for standby. Gastric lavage dose of 1 kg mice = Mg/60 kg × 9 (M refers to dose of Chinese medicine and 60 kg for adult standard weight). Ethanol gastric lavage dose is 2.2 g/kg (preexperiments were used to observe the effect of dose of 1.8 g/kg, 2.0 g/kg, 2.2 g/kg, and 2.4 g/kg; we found that 2.2 g/kg of ethanol has minimum effect on the spontaneous activity of mice).

### 2.3. Apparatus

Conditioned place preference experiment instrument: the box volume is 60 cm × 30 cm × 30 cm and middle of the box has 30 cm moveable partition which divided the box into two chambers with the same volume. After having taken out the partition, the mice can move freely between the two chambers. One side of the box body except glass panel was painted black, with a soft blanket to make the bottom surface rough. The other side was painted white except the glass panel and the bottom was smooth. The whole experiment box with two clues of visual and tactile is used to evaluate the rewarding effect of drugs and is an effective tool of finding antidrug-seeking behavior.

ZZ-6 independent activity tester (Chengdu Thai Union Technology Co., Ltd.): six grids spontaneous activity boxes measure spontaneous activity of six mice simultaneously, 36 infrared array probe devices with high resolution, and the function of analyzing the PC data collection. Materials are the double aluminum plates and sound and light insulation, with ventilation device. Animal activity status and times of spontaneous activity are recorded by infrared probe.

### 2.4. Agentias

Rat DA Elisa assay kit (Kit Item: E-30236), Rat GABA Elisa assay kit (Kit Item: E-30324) and Rat Glu Elisa assay kit (Kit Item: E-31033) are produced by Abcam company and imported and packaged by Beijing Yonghui Biotechnology Co., Ltd.

### 2.5. Experimental Methods

Behavioral sensitization test methods and procedures are detailed in Table 2.

*Habituation Phase (Day-3–Day-1).* In the case of not giving any medication, the animals were tested in the test chamber for 15 min. This procedure was repeated every day, during a 3-day period. The purpose is to let the KM mice adapt the test device, excluding the influence of environment and gastric administration on the spontaneous activity of mice and record their test baseline of spontaneous activity.

*Treatment Phase (Day1–Day10).* After 48 h of baseline measurement, 120 mice were randomly divided into 4 groups, half an hour in advance to be administered ASF or saline, followed by gavage saline or ethanol (2.2 g/kg). The four groups are saline + saline (S + S, *n* = 30), ASF + saline (Z + S, *n* = 30), saline + ethanol (S + E, *n* = 30), and ASF + alcohol group (Z + E, *n* = 30). The animals were tested in the test instrument for 15 min, immediately after ethanol (or saline) administration. This procedure was repeated every other day, during a 10-day period (five tests). Forty-eight hours after the end of this treatment, the challenge phase started.

*Challenge Phase (Day11).* After 48 hours of drug-free period, based on the last time results of spontaneous activity of mice from each large group, mice in the group can be randomly divided into three subgroups. The challenge phase included saline challenge and drug challenge.In saline challenge, the animals were tested in the test chamber for 15 min, immediately after saline administration. After 48 hours, the drug challenge began, immediately after three subgroups received ethanol, ASF, and ASF + ethanol, and the mice were tested in the spontaneous activity instrument for 15 min.

*Specimen Collection and Detection.* The mice of four subgroups (S + S + E, *n* = 10; S + E + E, *n* = 10; S + E + Z + E, *n* = 10; and Z + E + E, *n* = 10) were decapitated immediately after the test. The brain tissue was placed on the ice pillow after being dissected; the mesolimbic areas of the brain tissue were clipped and washed with ice-cold distilled water. Then put it in a 5 mL glass homogenizer prefilled with ice-cold saline and it is homogenated for 3 min, 5000 rpm freezing centrifuged for 10 min, and the supernatant was collected. The specific procedures of Elisa method have 11 steps, which include standard dilution, sample adding, incubation, liquid mixing, enzyme, incubation, washing, coloration, termination, zero adjustment, and measuring the absorbance at 450 nm wavelength (OD). Measurement should be carried out within 15 minutes after the stop solution.

**Figure 1:**
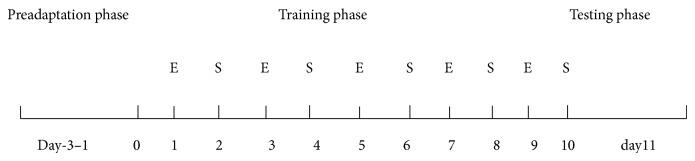
Timeline of CPP procedure.

*Conditioned Place Preference Experiment.* In this study, the experimental procedure is biased. Experiment was divided into preadaptation phase, training phase, and expressing testing. Light, color, odor, and other environmental conditions in the box are consistent throughout the experiment (Figure 1).

*Preadaptation Phase (Day-3–Day-1).* The animals were placed in the middle of the CPP box and allowed free movement for 15 min. This procedure was repeated every day, during a 3-day period and saline was administrated every day to eliminate the effect of the experimental operation on mice. Residence time of mice in three boxes in the 3rd day was recorded. The residence time of the mice in different regions within 15 min was recorded as index of natural preference. The long-time side of the box was nonmedicine box and the other side was medicine box. Under the condition of this experiment, mice had a natural preference of black. So we adopted experimental design with bias and used white chamber as medicine chamber and black chamber as nonmedicine chamber.

*Training Phase (Day1–Day10).* After preadaptation phase, the animals were randomized into 4 groups, 12 in each group (saline: S + S, ethanol: S + E, ASF: Z + S, and ASF + ethanol: Z + E). On odd-numbered days, animals were administered (i.g.) saline or ASF (8.1 g/kg/d) and then administered (i.g.) saline or ethanol (2.2 g/kg) after 30 min and placed in medicine box for 1hour immediately. On even-numbered days, animals were administered (i.g.) saline before being placed in nonmedicine box for training. There are 5 medicine/saline training cycles in total. The training time is fixed between 8 points and 9 points every morning.

*Testing Phase (D11).* The animals were placed in middle box for 15 min half an hour after saline is administered. The residence time of mice in medicine box and nonmedicine box was recorded.

*Statistical Treatment.* All variables are expressed as $\overset{-}{x}\pm \text{S}.\text{E}.\text{M}$. The statistical analysis was carried out by the SPSS 17.0 software. The data between two groups were compared using Student's *t*-test data; the single-factor analysis of variance was used in multiple sets of quantitative data means comparison, and SNK method (student-Newman-Keuls) was used in multiple comparisons; with time variable data, the single factor analysis of variance for repeated measurements was used and Bonferroni post tests method were used for multiple comparisons. The times of challenge phase activity were compared using analysis of covariance (ANCOVA) and LSD method for multiple comparisons. CPP in mice in medicine box of before and after comparison used a paired Student's *t*-test. Means of multiple groups were compared with analysis of one-way ANOVA. LSD was used for pairwise comparison. All *P* values are two-sided test. The significance level was set at 5%.

## 3. Results

### 3.1. Results of Behavioral Sensitization

#### 3.1.1. The Baseline Locomotor Activity of Mice in the Habituation Test (Figure 2)

One-way ANOVA showed no statistically significant difference of mice baseline locomotor activity in the habituation test, according to post hoc LSD test.

#### 3.1.2. Effect of Repeated Dose on Spontaneous Activity of Mice (Figure 3)

**Figure 3:**
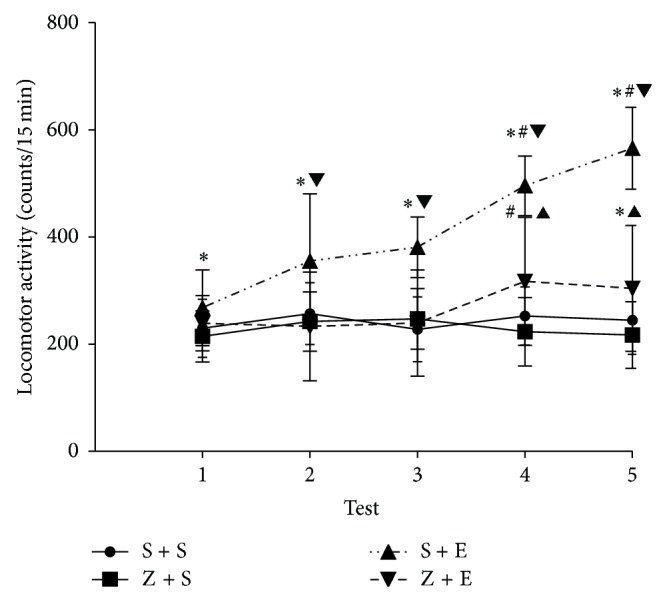
Spontaneous activity (mean ± SEM, counts in 15 min) in the 5 tests during the 10-day period of treatment, immediately after ethanol or saline administration. Mice were pretreated with ASF or saline 30 mins before the test (data were analysed by One-way repeat measures ANOVA and followed by Bonferroni posttest). Groups: saline + saline (S + S, *n* = 30), ASF + saline (Z + S, *n* = 30), saline + ethanol 2.2 g/kg (S + E, *n* = 30), and ASF + ethanol 2.2 g/kg (Z + E, *n* = 30). ^*^Higher levels than S + S group in tests 1, 2, 3, 4, and 5 (*P* < 0.01). ^#^Higher activity levels than in tests 1 2, and 3 (*P* < 0.05), ^▲^Z + E group Higher levels than S + S group, Z + S group in tests 4 and 5 (*P* < 0.05), ^▼^S + E group Higher levels than Z + S group in 2, 3, 4, and 5 tests (*P* < 0.01).

Figure 3 shows the locomotor activity of mice during five tests 10-day experiment with repeated administration of ethanol (2.2 g/kg), ASF (8.1 g/kg), or ASF + ethanol. One-way ANOVA for repeated measures detected significant effects of time factor [*F* = 42.3; *P* < 0.01]; treatment factor [*F* = 138.7; *P* < 0.01]; and time × treatment factor [*F* = 22.7; *P* < 0.01]. The Newman-Keuls test showed significantly higher locomotor activity levels in the S + E treated group than in the S + S group, during the 5 tests (*P* < 0.01). In the 2, 3, 4, 5, tests, the S + E treated group presented higher locomotor activity than Z + E treated group and (*P* < 0.01) suggested that the administration of ASF (8.1 g/kg) 30 min before the ethanol could reduce high locomotor activity of ethanol induced mice. The Newman-Keuls post hoc test also detected no statistically difference when compared with the S + S treated group and Z + S treated group in 5 tests (*P* > 0.05) and implied that the ASF did not affect the locomotor activity of mice.

#### 3.1.3. The Effect of ASF on the Behavioral Sensitizations Induced by Ethanol in Mice (Figure 4)

**Figure 4:**
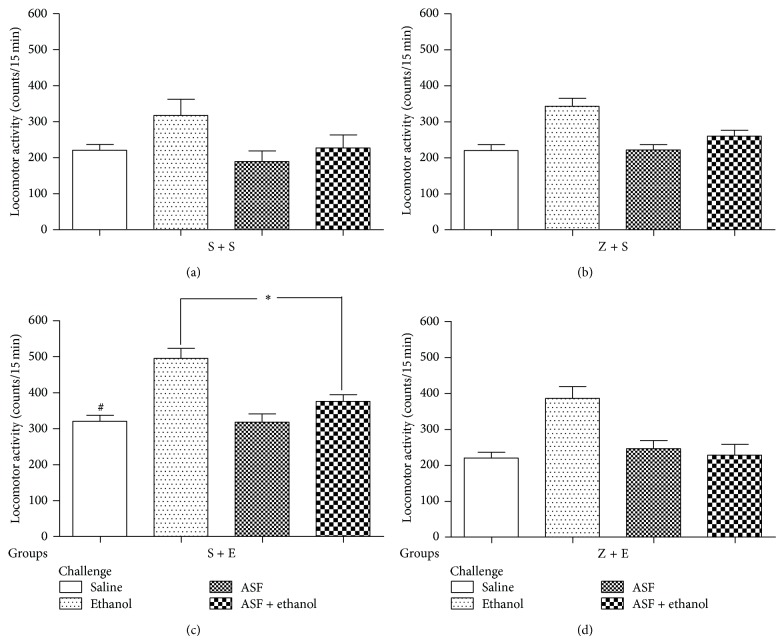
Spontaneous activity (mean ± SEM, counts in 15 min) in challenge tests of mice previously treated with (a) saline + saline (S + S); (b) 8.1 g/kg of ASF + saline (Z + S); (c) saline + 2.2 g/kg of ethanol (S + E); and (d) 8.1 g/kg of ASF + 2.2 g/kg of ethanol (Z + E). All animals were tested under saline + saline (saline challenge) with 30 min of interval between saline and saline administration “Drug challenge” was assigned 48 h after “saline challenge.” Different animals were used in each “Drug challenge.” Saline + 2.2 g/kg of ethanol (ethanol; *n* = 10); saline + ASF 8.1 g/kg (ASF; *n* = 10); and ASF 8.1 g/kg + ethanol 2.2 g/kg (ASF + ethanol; *n* = 10). The mice were tested in the spontaneous activity cages for 15 min immediately after ethanol (or saline) administration. ^#^Higher activity levels than those from all other treatment groups in the saline challenge (*P* < 0.05). ^*^Higher spontaneous activity levels than all the other groups, in all drug challenges (*P* < 0.01).

From Figure 4, we can see the locomotor activity of the pretreated group (S + S, S + E, Z + S, and Z + E) after the saline and drug challenges (saline, ASF, ethanol, and ASF + ethanol). The S + E pretreated group showed significantly higher locomotor activity levels than all other groups (*P* < 0.01), followed by A one-way ANOVA LSD test. Indicating mice behavioral sensitization model to ethanol had been built successfully. In the S + E pretreated group, we can observe the administration of ASF (8.1 g/kg) 30 min before ethanol challenge block the ethanol effect (*P* < 0.01). The result suggests that the ASF can block the expression of behavior sensitization induced by ethanol. We also investigated that ASF (8.1 g/kg) blocked the development of sensitization to ethanol. In the Z+E pretreated group, we found that ethanol induced locomotor activity in the ASF preexposed group is not significantly different than in the other groups (*P* > 0.05). It states that the ASF prevents the development of behavior sensitization induced by ethanol in mice.

#### 3.1.4. Effect of ASF on DA, Glu, and GABA Levels in the Brain Tissue of Mice (Figure 5)

The concentration of DA and Glu of S + E + E group was higher than that of S + S + E group (*P* < 0.01). Compared with the ethanol group, the ASF group had low level of DA and Glu (*P* < 0.01). In the inhibitory GABA three group comparisons, compared with the saline control group, S + E + E group's GABA decreased significantly (*P* < 0.01), and compared with the ethanol group, the ASF group increased the release of GABA.

### 3.2. Results of CPP Experiment

#### 3.2.1. Natural Preference Effect Experiments in Mice (Figure 6)

The residence time of mice in black chamber was 579.97 ± 23.634 s in preexperiment while the time in white chamber was 277.75 ± 67.686 (*P* < 0.01). The result suggested that mice had natural preference of black sides, metal bottom grid box. Therefore, we adopted experimental design with bias and used white chamber as medicine chamber and black chamber as nonmedicine chamber.

#### 3.2.2. Effect on ASF of Mice Induced by Ethanol in CPP Development Phase (Figure 7)

**Figure 7:**
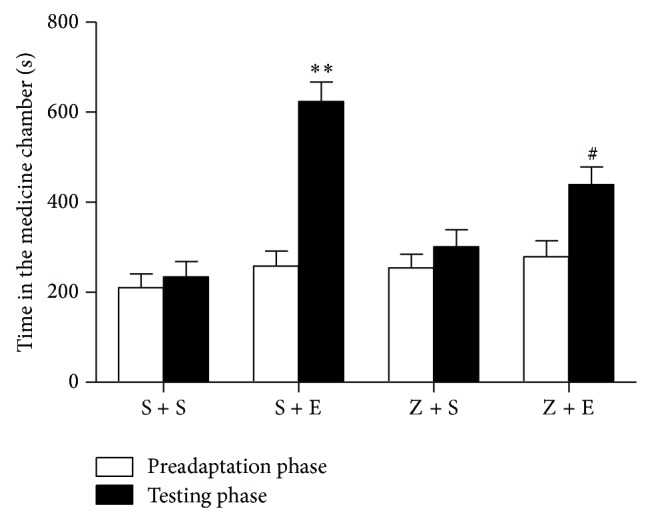
Effect of ASF on development of ethanol-induced CPP in mice (mean ± SEM, *n* = 12), ^**^ *P* < 0.01 versus NS group, ^#^ *P* < 0.05 versus ethanol group (a paired Student's *t*-test).

After a 10-day period (five tests), time spent in medicine chamber (623.8 s) of S + E group in mice was higher than that of S + S group (234.0 s) (*P* < 0.001). The time spent in medicine chamber of Z + S group (*n* = 12) was 438.7 s. The time spent of Z + E group in medicine chamber was shorter than that of S + E group in nonmedicine group (*P* < 0.05) (Figure 7).

## 4. Discussion

Behavioral sensitization performance is characterized by an increase in spontaneous activity after continuous use of addictive drugs [21]. Behavioral sensitization can be seen in use of cocaine, morphine, and nicotine which is a key part of drug addiction [22–24]. According to drug addiction motivation sensitization theory, behavioral sensitization plays an important role in forced medication, drug-seeking behavior, and behavior of relapse after withdrawal [25]. The main mechanism of behavioral sensitization is adaptation and synaptic plasticity in the central nervous system [26], which is a recognized model to study drug addiction [23, 24]. Repeated activation of mesolimbic dopamine system (MDLS) and release of increasing rewarding effects of alcohol are considered the key to the formation of behavioral sensitization. Study found that alcohol can increase concentrations of DA of NAc [27], and the release of DA after the withdrawal of alcohol also decreased [28]. Experiments have shown that, in the process of the formation of behavioral sensitization, there is an increase of Glu in the prefrontal cortex, amygdala, hippocampus, other limbic brain regions, and VTA [29]. Evidence shows that excitatory pathway mediated by glutamate plays an important role in the pathogenesis of alcohol dependence, and the use of drugs against glutamate as acamprosate has effect in treatment of alcohol addiction [30]. GABA is the major inhibitory neurotransmitter in central nervous system. It inhibits the release of DA of mesolimbic to weaken the effect of cocaine, heroin, and nicotine, alcohol, and other addictive drugs [31]. CGP7930 (GABAB receptor positive allosteric modulator) can reduce the motivation of animal seeking for alcohol and obviously delay the pedal time, the first time the rat seek for alcohol [32].

CPP is a method based on classical conditioning to evaluate the reinforcing effect of drug. After the repeated contact of reward stimulation and a specific environment (nonreward neutral stimulus), the latter will get reward properties. A certain environment can induce nonconditioned behavioral response associated with nonconditioned reward and this phenomenon is “response reinforcement.” CPP is a classic experiment based on this theory to determinate material reward and spiritual dependence, which shows the natural preference side change after drug training. Morphine and amphetamine can induce CPP in rat. However, there are few research reports at home and abroad about alcohol-induced CPP in mice.

In this study, we used ethanol induced behavioral sensitization in mice and CPP model as the research objects and tested the intervention of ASF in sensitization in mice and CPP formation. In behavioral sensitization experiments, mice were administrated ethanol in advance, and only the mice induced by ethanol showed behavioral sensitization (times of spontaneous activity increase). Mice that are intragastric administrated ASF + ethanol and induced by ethanol and mice that are administrated ethanol and induced by ASF + ethanol showed no behavioral sensitization. And the concentration of DA and Glu from mesolimbic region obviously decreased compared with mice administrated ethanol and induced by ethanol, but the concentration of GABA increased significantly, which indicated that ASF can inhibit the expression of behavioral sensitization induced by ethanol in mice by increasing the content of GABA and decreasing the contents of DA and Glu of sensitized brains of mice. The mice that are administrated ASF and induced by ASF showed no significantly increase or decrease in spontaneous activity, which indicated that ASF itself does not induce sensitization in mice. And it reduced the times of spontaneous activity in mice induced by ethanol which was not because of the nonspecific sedative effect of it, but because it could interfere with the stimulating effect of ethanol on the central nervous system.

CPP test is a method of evaluating the rewarding effects of drugs and potential of psychological dependence. In a way, CPP reflects drug-seeking behavior in animals and the psychological craving in humans [33]. The CPP test found that the white box residence time of ethanol groups of trained mice was longer than time of mice before training and significantly higher than those in the saline groups, which reflects the psychological dependence of alcohol. The residence time of mice from saline + ASF group in white box had no significant differences before or after training, which explained that ASF itself does not induce the formation of CPP in mice and does not have a psychological dependence. The residence time of mice from ASF + ethanol group in white box had no significant differences before or after training, which indicated that ASF inhibits the formation of CPP induced by ethanol in mice.

In summary, the study found that ASF can inhibit development and expression of behavioral sensitization induced by ethanol and the development of CPP in mice. We demonstrate the inhibition of ASF on behavioral sensitization partly due to its affect on the mesolimbic neurotransmitter system, including decreasing level of DA, Glu and increasing the content of GABA. Thus, we can infer that ASF can prevent the forced medication behavior, drug-seeking behavior after withdrawal, and the relapse behavior of alcohol addicts and has intervention effects of alcohol dependence. This study provides a certain experimental basis for the intervention of alcohol addiction mechanism of ASF, but the other mechanisms of ASF's effect on antialcohol addiction need further study.

## Figures and Tables

**Figure 2:**
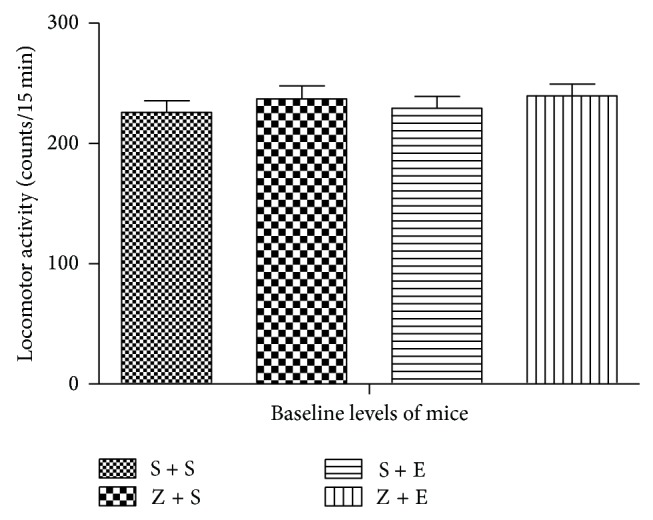
The baseline of locomotor activity (mean ± SEM, counts in 15 min) in the habituation test. There is no difference in which mice were divided into four groups: saline + saline (S + S, *n* = 30), ASF + saline (Z + S, *n* = 30), saline + ethanol (S + E, *n* = 30), and ASF + ethanol (Z + E, *n* = 30). (*P* > 0.05).

**Figure 5:**
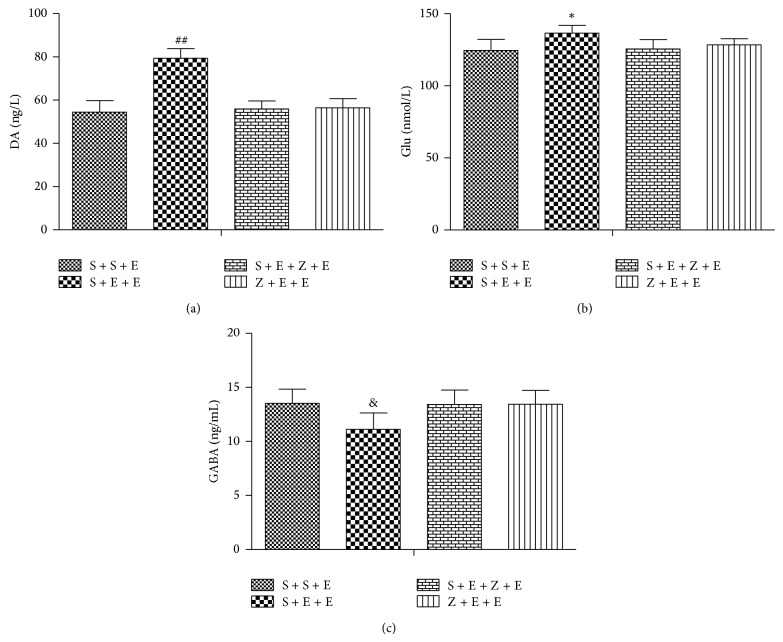
^**^ *P* < 0.01. versus other three groups (a). ^#^ *P* < 0.05 versus other three groups (b), ^&^ *P* < 0.05 versus other three groups (c), the levels (ng/L) of DA, the levels (nmol/L) of Glu, and the levels (ng/mL) of GABA are presented as mean ± SEM, by one-way ANOVA followed by a post hoc LSD test.

**Figure 6:**
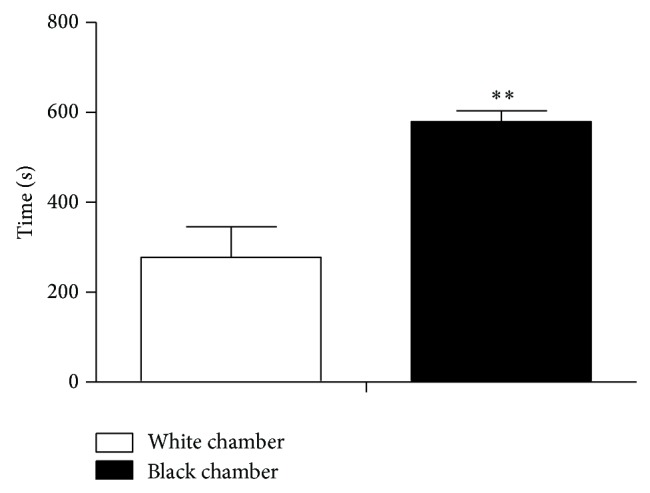
Time spent in the white and black chambers in a pretest before training phase. ^**^ *P* < 0.001 compared with the white chamber. The values are expressed as the mean ± SEM (*n* = 12), by unpaired *t* test.

**Table 1:** The formula of ASF (one dose).

Herb	Medicinal parts	Origin	Amount in preparation (g)
Ziziphus jujuba			
Mill. var. spinosa	Fruit	Hebei Province	30
Epimedium brevicornu	Rhizoma and Leaf	Shanxi Province	24

**Table 2:** Experimental groups and treatments. Pretreatment drug administration (i.g.) was given 30 min before treatment.

Group	Treatment phase		Challenge phase			
	Days 1, 3, 5, 7, and 9		Day 11 (saline challenge)		Day 13 (drug challenge)	
	Pretreatment	Treatment	Pretreatment	Treatment	Pretreatment	Treatment
S + S	Saline	Saline	Saline	Saline	Saline	Ethanol
					ASF	Saline
					ASF	Ethanol

Z + S	ASF	Saline	Saline	Saline	Saline	Ethanol
					ASF	Saline
					ASF	Ethanol

S + E	Saline	Ethanol	Saline	Saline	Saline	Ethanol
					ASF	Saline
					ASF	Ethanol

Z + E	ASF	Ethanol	Saline	Saline	Saline	Ethanol
					ASF	Saline
					ASF	Ethanol

## References

[1] Volpicelli J. R., Volpicelli L. A., O'Brien C. P. (1995). Medical management of alcohol dependence: clinical use and limitations of naltrexone treatment. *Alcohol and Alcoholism*.

[2] San L., Pomarol G., Peri J. M., Olle J. M., Cami J. (1991). Follow-up after a six-month maintenance period on naltrexone versus placebo in heroin addicts. *British Journal of Addiction*.

[3] Shufman E. N., Porat S., Witztum E., Gandacu D., Bar-Hamburger R., Ginath Y. (1994). The efficacy of Naltrexone in preventing reabuse of heroin after detoxification. *Biological Psychiatry*.

[4] Kranzler H. R., Burleson J. A., Korner P., Del Boca F. K., Bohn M. J., Brown J., Liebowitz N. (1995). Placebo-controlled trial of fluoxetine as an adjunct to relapse prevention in alcoholics. *The American Journal of Psychiatry*.

[5] Johnsen J., Stowell A., Morland J. (1992). Clinical response in relation to blood acetaldehyde levels. *Pharmacology and Toxicology*.

[6] Johnson B. A. (2008). Update on neuropharmacological treatments for alcoholism: scientific basis and clinical findings. *Biochemical Pharmacology*.

[7] Quertemont E. (2004). Genetic polymorphism in ethanol metabolism: acetaldehyde contribution to alcohol abuse and alcoholism. *Molecular Psychiatry*.

[8] Quertemont E., Grant K. A., Correa M., Arizzi M. N., Salamone J. D., Tambour S., Aragon C. M. G., McBride W. J., Rodd Z. A., Goldstein A., Zaffaroni A., Li T.-K., Pisano M., Diana M. (2005). The role of acetaldehyde in the central effects of ethanol. *Alcoholism: Clinical and Experimental Research*.

[9] Keung W. M., Vallee B. L. (1993). Daidzin: a potent, selective inhibitor of human mitochondrial aldehyde dehydrogenase. *Proceedings of the National Academy of Sciences of the United States of America*.

[10] Keung W.-M., Vallee B. L. (1993). Daidzin and daidzein suppress free-choice ethanol intake by Syrian golden hamsters. *Proceedings of the National Academy of Sciences of the United States of America*.

[11] Keung W. M., Lazo O., Kunze L., Vallee B. L. (1995). Daidzin suppresses ethanol consumption by Syrian golden hamsters without blocking acetaldehyde metabolism. *Proceedings of the National Academy of Sciences of the United States of America*.

[12] Guoyuan C., Chong L., Jianying Z. (2010). Three -year follow-up research of the alcohol dependence patients who used traditional Chinese Medicine(alcohol detoxification soup) or furazolidone. *China Journal of Health Psychology*.

[13] Hong D. U. (2011). Treating 520 cases of alcohol dependence with Jiecheng oral solution. *Journal of Practical Traditional Chinese Internal Medicine*.

[14] Ma J., Liu P., Ma B. (2011). The chemical constituents of Semen Zizyphi Spinosae and pharmacologic mechanism of its sedative and hypnotic effects: research advances. *Journal of International Pharmaceutical Research*.

[15] Zhang M., Ning G., Shou C., Lu Y., Hong D., Zheng X. (2003). Inhibitory effect of jujuboside A on glutamate-mediated excitatory signal pathway in hippocampus. *Planta Medica*.

[16] Fu L.-B., Xia Y.-H., Yu L. (2007). Experimental study of Epimedium total flavonoids on arterial pressure in rats and its mechanism. *Chinese Journal of Applied Physiology*.

[17] Broadbent J., Weitemier A. Z. (1999). Dizocilpine (MK-801) prevents the development of sensitization to ethanol in DBA/2J mice. *Alcohol and Alcoholism*.

[18] Phillips T. J., Lessov C. N., Harland R. D., Mitchell S. R. (1996). Evaluation of potential genetic associations between ethanol tolerance and sensitization in BXD/Ty recombinant inbred mice. *Journal of Pharmacology and Experimental Therapeutics*.

[19] Fish E. W., DeBold J. F., Miczek K. A. (2002). Repeated alcohol: Behavioral sensitization and alcohol-heightened aggression in mice. *Psychopharmacology*.

[20] Newlin D. B., Thomson J. B. (1999). Chronic tolerance and sensitization to alcohol in sons of alcoholics: II. Replication and reanalysis. *Experimental and Clinical Psychopharmacology*.

[21] Robinson T. E., Becker J. B. (1986). Enduring changes in brain and behavior produced by chronic amphetamine administration: a review and evaluation of animal models of amphetamine psychosis. *Brain Research Reviews*.

[22] Hunt W. A., Lands W. E. M. (1992). A role for behavioral sensitization in uncontrolled ethanol intake. *Alcohol*.

[23] Robinson T. E., Berridge K. C. (1993). The neural basis of drug craving: an incentive-sensitization theory of addiction. *Brain Research Reviews*.

[24] Meredith G. E., Pennartz C. M., Groenewegen H. J. (1993). The cellular framework for chemical signalling in the nucleus accumbens. *Progress in Brain Research*.

[25] Meredith G. E., Pennartz C. M. A., Groenewegen H. J. (1993). The cellular framework for chemical signalling in the nucleus accumbens. *Progress in Brain Research*.

[26] Ikemoto M., Takita M., Imamura T., Inoue K. (2000). Increased sensitivity to the stimulant effects of morphine conferred by anti-adhesive glycoprotein SPARC in amygdala. *Nature Medicine*.

[27] Yim H. J., Gonzales R. A. (2000). Ethanol-induced increases in dopamine extracellular concentration in rat nucleus accumbens are accounted for by increased release and not uptake inhibition. *Alcohol*.

[28] Brodie M. S., Pesold C., Appel S. B. (1999). Ethanol directly excites dopaminergic ventral tegmental area reward neurons. *Alcoholism: Clinical and Experimental Research*.

[29] Nakagawa T., Kaneko S. (2008). Neuropsychotoxicity of abused drugs: Molecular and neural mechanisms of neuropsychotoxicity induced by methamphetamine, 3,4- methylenedioxymethamphetamine (ecstasy), and 5-methoxy-N,N-diisopropyltryptamine (foxy). *Journal of Pharmacological Sciences*.

[30] Rao P. S. S., Sari Y. (2012). Glutamate transporter 1: target for the treatment of alcohol dependence. *Current Medicinal Chemistry*.

[31] Dalley J. W., Everitt B. J. (2009). Dopamine receptors in the learning, memory and drug reward circuitry. *Seminars in Cell and Developmental Biology*.

[32] Liang J. H., Chen F., Krstew E., Cowen M. S., Carroll F. Y., Crawford D., Beart P. M., Lawrence A. J. (2006). The GABAB receptor allosteric modulator CGP7930, like baclofen, reduces operant self-administration of ethanol in alcohol-preferring rats. *Neuropharmacology*.

[33] Liu Y., Le Foll B., Wang X., Lu L. (2008). Conditioned place preference induced by licit drugs: establishment, extinction, and reinstatement. *The Scientific World Journal*.

